# The contribution of S100B to the glioprotective effects of valproic and arundic acids

**DOI:** 10.22038/ijbms.2019.29852.7204

**Published:** 2019-05

**Authors:** Mojtaba Keshavarz, Majid Reza Farrokhi, Atena Amiri, Mahshid Hosseini

**Affiliations:** 1 Shiraz Neuroscience Research Center, Shiraz University of Medical Sciences, Shiraz, Iran

**Keywords:** Amyloid-β-peptide, Arundic acid, Astrocytes, S100B, Valproic Acid

## Abstract

**Objective(s):**

Valproic and arundic acids are astrocytes-modulating agents with potential effects in the treatment of Alzheimer’s disease (AD). S100B is an astrocytic cytokine with a possible role in the pathogenesis of AD. In this study, we aimed to assess the glioprotective effects of valproic and arundic acids against amyloid-β-peptide (Aβ)-induced glial death and contribution of S100B to the glioprotective effects of these agents in an astrocytic culture.

**Materials and Methods:**

We used Aβ25–35 at a concentration of 200 μM in 1321N1 astrocyte cells. We treated the cells with valproic acid (0.5 and 1 mM) and/or arundic acid 50 µM for 24 hr. Methylthiazolyldiphenyl-tetrazolium bromide (MTT) test was used to measure cell viability. The intracellular and extracellular S100B levels were measured using an ELISA kit. The data were analyzed using one-way analysis of variance followed by the Tukey’s test.

**Results:**

Aβ (200 µM) decreased the cell viability compared to the control group (*P*<0.001). Valproic acid (0.5 and 1 mM) and arundic acid (50 µM) ameliorated the gliotoxic effects of Aβ (*P*<0.05). The Aβ-treated group had higher S100B levels (both intracellular and extracellular) compared to the negative control groups (*P*<0.001). Arundic and valproic acids (0.5 and 1 mM) decreased both the intracellular and extracellular S100B levels compared to the Aβ-treated group (*P*<0.001).

**Conclusion:**

By considering homeostatic and neuroprotective functions of astrocyte, the astroprotective effects and the attenuation of S100B level may be responsible, at least in part, for the beneficial effects of valproic and arundic acids in AD.

## Introduction

Alzheimer’s disease (AD) is a progressive neurodegenerative disorder which affects several brain regions responsible for learning and memory (1). The main pathological findings are the aggregation of amyloid-β-peptide (Aβ) and the formation of neurofibrillary tangles (2). Recently, convincing evidence has also indicated the contribution of glial dysfunction or loss in the pathogenesis of AD (3). 

Astrocytes are the most prevalent glial cells with probable roles in the pathophysiology of AD. *In vitro* and *in vivo* studies have shown the close association of Aβ with astrocytes (4, 5). Astrocytes internalize and degrade Aβ and prevent the aggregation of Aβ extracellular plagues (6, 7). The aggregation of Aβ in astrocytes may cause astrocytic lysis and lead to astrocytic plaques (8). The progressive loss of astrocytic functions like energy metabolism (9), glutamate recycling (10), and glutathione supply (11, 12) may affect homeostatic and neuroprotective functions of these cells and promote the neurodegenerative process (13, 14). Thus, a potential cure for the treatment of AD may be restoration of the astrocytic function to amend the homeostatic process and avoid neurodegeneration (15). 

Valproic and arundic acids are potential drugs for the treatment of AD (16). Some evidence shows that astrocytes may be the primary target of these two agents (17, 18). Arundic acid is a new derivative of valproic acid with inhibitory effects on the synthesis and release of S100B in astrocytes (19). By considering homeostatic functions of astrocytes in the central nervous system (CNS), protection of astrocytes by modulation of S100B may be relevant to the arundic acid mechanism of action. Moreover, the glioprotective effects of valproic acid and the contribution of S100B suppression to its glioprotective effects are elusive. Thus, we aimed to assess the glioprotective effects of valproic and arundic acids against Aβ-induced glial death. Moreover, we also aimed to explore the contribution of S100B to the glioprotective effects of these agents in the 1321N1 astrocyte culture. 

## Materials and Methods

***Materials***

1321N1 astrocyte cells were purchased from the Pasteur Institute (Tehran, Iran). Cell culture materials including DMEM/F12, FBS (fetal bovine serum), and penicillin-streptomycin were obtained from Gibco® life technologies™ (New York, USA). We obtained Aβ, valproic acid, methylthiazolyldiphenyl-tetrazolium bromide (MTT), phosphate buffered solutions (PBS), and dimethyl sulfoxide (DMSO) from the Sigma-Aldrich Corporation (St. Louis, USA). We purchased arundic acid (ONO-2506) from Tocris Bioscience (USA). 

***Astrocyte cell culture***

We kept 1321Na cells in a t75 flask and maintained it at 37 ^°^C in 95% humidified atmosphere/5% CO_2_. Upon reaching confluency, we detached the cells from the flask by 0.25% Trypsin/EDTA. We then centrifuged the cell suspension for 10 min at 1200 rpm and 22 ^°^C. The cells then were seeded on 6 or 96 well plates. 

***Aβ25–35 preparation***

Aβ25–35 at a concentration of 2 μg/μl was dissolved in sterile distilled water and kept in the freezer (at −70 ^°^C) until use. Aβ25–35 was aggregated by incubation for 4 days at 37 ^°^C before administration in the cell culture. 

***Treatments***

We dissolved Aβ and valproic acid in PBS and arundic acid in a solution of PBS and DMSO (5% v/v). We calculated the effective concentration of Aβ25-35, valproic acid, and arundic acid by dose-response experiments and MTT assay. On the day of the experiment, we treated the cells with the chosen concentrations of each agent. We incubated the cells with Aβ (200 μM), valproic acid (0.5 and 1 mM), and arundic acid (50 µM), or both for 24 hr. 

***Cell viability assay***

We used MTT reagent to measure cell viability, and added 5 mg/ml MTT reagent to the cell culture media 24 hr after the treatments. Four hours later, we removed the cell culture media and dissolved the precipitation of each well in 100 μl of DMSO. Then, we measured the absorbance of each well at 570 nm by a microplate reader (Synergy HT, Biotek®).

***Sample preparation for measurement of the S100B protein***

After the dose selection, astrocytes were cultured in the 6-well plates in a condition similar to the cell viability assay. The intracellular S100B level was measured using the astrocytic cell lysate. We used pre-cooled PBS and trypsin to detach cells from the plates. The suspended cells were centrifuged for 5 min at 1000×g and then lysed with three cycles of freeze-thawing. Then the cell mixture was centrifuged at 1500×g, 4 ^°^C. The cell culture supernatants were centrifuged at 1000×g and 4 ^°^C and used for measurement of the extracellular level of S100B. 

***Measurement of S100B level***

Twenty-four hours after the treatment, intracellular and extracellular S100B levels were measured using a commercially available ELISA kit (Mybiosource Inc., USA). The experimental procedure was compatible with the manufacturer’s instructions. In brief, 100 μL of the standard or samples were added to each well and incubated for 90 min at 37 ^°^C. Then, 100 μl of the biotinylated detection Ab was added to the separated supernatant and incubated for 1 hr at 37 ^°^C. After washing, 100 μl of the HRP conjugate was added and incubated for 30 min at 37 ^°^C. After washing, we poured the substrate reagent into wells and incubated for 15 min at 37 ^°^C. Finally, we poured the stop solution (50 μL) into each well and measured the absorbance at 450 nm using a microplate reader (Synergy HT, Biotek®). The concentration of the S100B protein was determined by a standard curve. 

***Statistical analysis***

The data were analyzed using the one-way analysis of variance (ANOVA) followed by the Tukey’s test. *P*<0.05 was considered as significance cutoff. All analyses were performed using the SPSS software, version 23. 

## Results

***Effects of different treatments on the glial cell viability***

**Figure 1:**
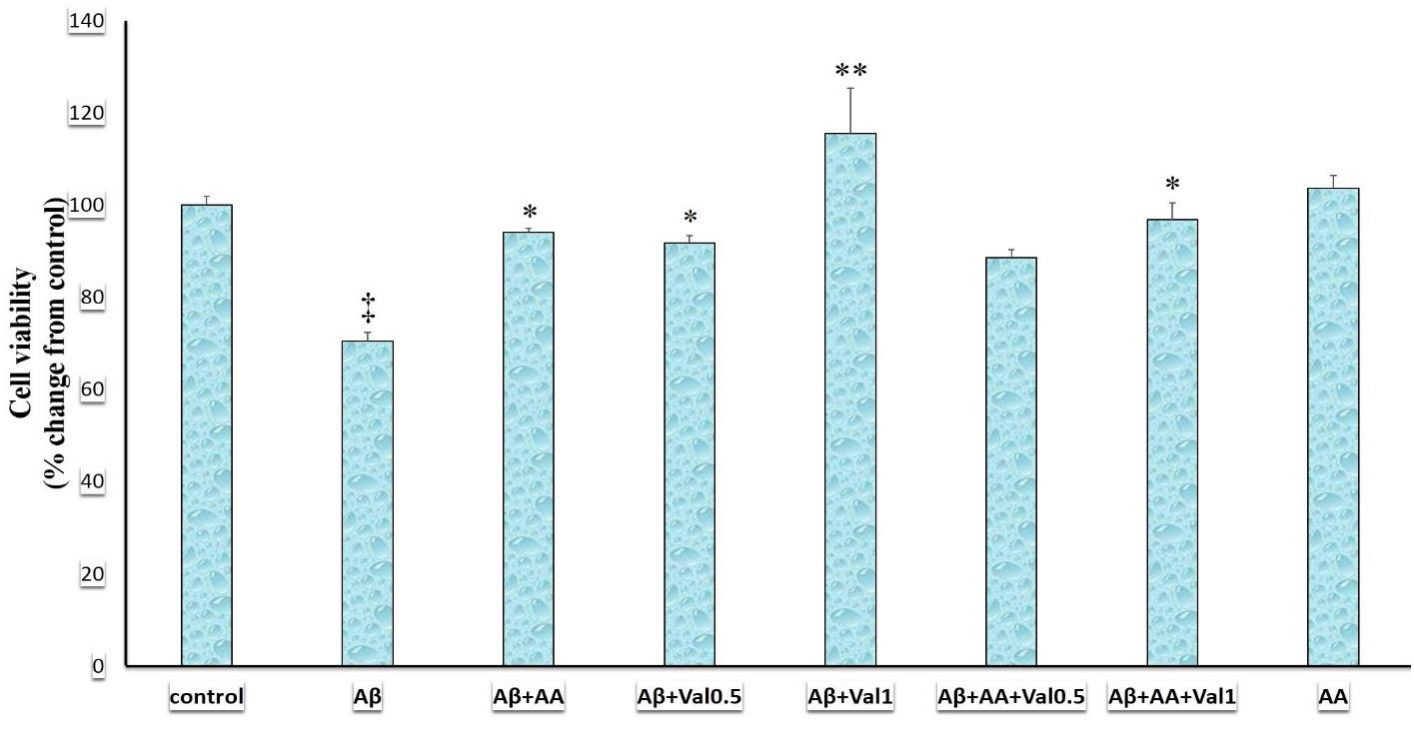
Cell viability in 1321N1 astrocytic cells exposed to the beta-amyloid (Aβ), valproic acid, and arundic acid. Cell viability was measured using the methylthiazolyldiphenyl-tetrazolium bromide (MTT) test. Data were analyzed using one-way analysis of variance (ANOVA) followed by the Tukey’s test. P-value of lower than 0.05 was the significant level. Aβ: beta-amyloid peptide25-35 (200 µM), AA: arundic acid (50 µM), Val0.5: valproic acid (0.5 mM), Val1: valproic acid (1 mM). ‡: *P*-value of <0.001 compared to the control, *, and **: *P*-value of <0.05 and <0.001 compared to the Aβ-treated group, respectively

This study showed that cell viability was different in various treatment groups (F (7, 24) = 10.139, *P*<0.001) (Figure 1). The administration of Aβ (200 µM) decreased the cell viability compared to the control group (*P*<0.001) (Figure 1). Addition of valproic acid (0.5 mM, *P*=0.021), valproic acid (1 mM, *P*<0.001), or arundic acid (50 µM, *P*=0.008) to Aβ ameliorated the gliotoxic effects of Aβ (Figure 1). Addition of arundic acid + valproic acid (1 mM) to Aβ also diminished the cell toxicity of Aβ (*P*<0.001) (Figure 1). However, the addition of arundic acid + valproic acid (0.5 mM) to Aβ had no significant effect on the Aβ-induced gliotoxicity. Arundic acid (50 µM, *P*=0.998) in the absence of Aβ exerted no effect on the survival of glial cells.

**Figure 2:**
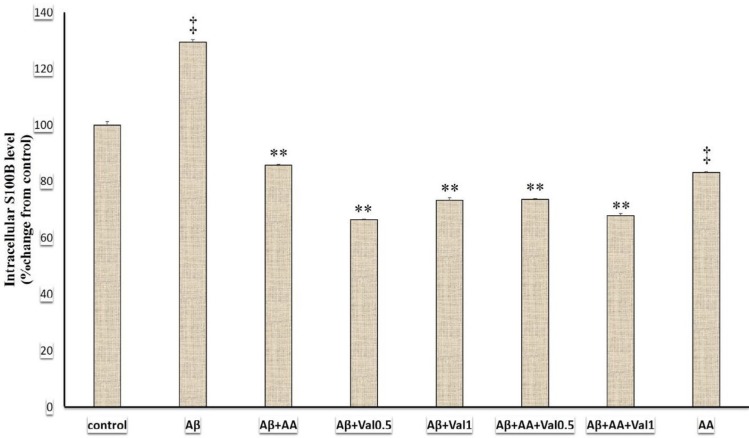
The intracellular S100B levels of 1321N1 astrocytic cells exposed to beta-amyloid (Aβ), valproic and arundic acids. The S100B concentration was measured in cell lysate via the ELISA method. Data were analyzed using one-way analysis of variance (ANOVA) followed by the Tukey’s test. *P*-value of lower than 0.05 was the significant level. Aβ: beta-amyloid peptide25-35 (200 µM), AA: arundic acid (50 µM), Val0.5: valproic acid (0.5 mM), Val1: valproic acid (1 mM). **: *P*-value of <0.001 compared to the beta-amyloid-treated group and ‡: *P*-value of <0.001 compared to the control-treated group

**Figure 3:**
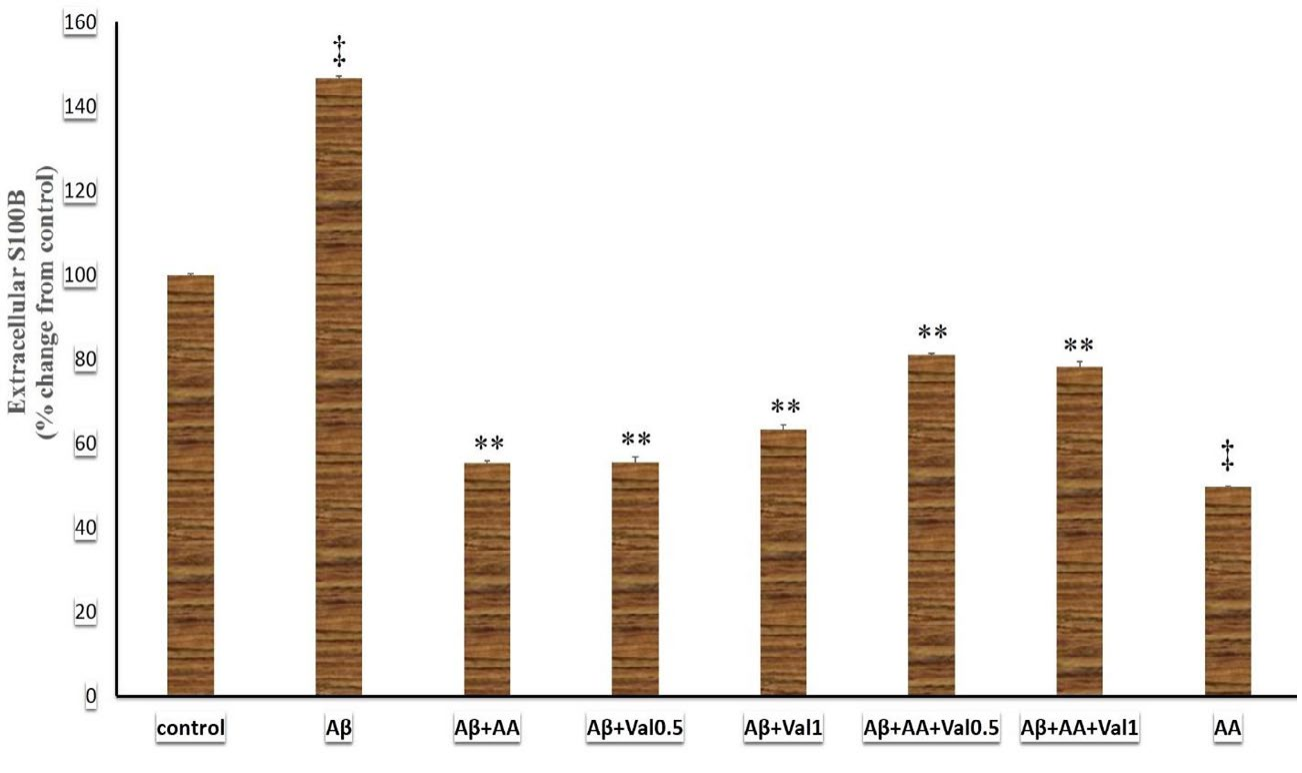
The extracellular S100B levels of 1321N1 astrocytic cells exposed to beta-amyloid, valproic acid, and arundic acid. The S100B concentration was measured in cell culture media via the ELISA method. Data were analyzed using one-way analysis of variance (ANOVA) followed by the Tukey’s test. *P*-value of lower than 0.05 was the significant level. Aβ: beta-amyloid peptide25-35 (200 µM), AA: arundic acid (50 µM), Val0.5: valproic acid (0.5 mM), Val1: valproic acid (1 mM). **:* P*-value of <0.001 compared to the beta-amyloid-treated group and ‡: *P*-value of <0.001 compared to the control-treated group

***Effects of different treatments on the intracellular S100B levels***

In this study, various treatments altered the intracellular S100B levels (F (7, 24) =988.95, *P*<0.001) (Figure 2). The Aβ-treated group had a significantly higher S100B level compared to the control group (*P*<0.001). The treatment with arundic acid suppressed the S100B level compared to the Aβ and control groups (*P*<0.001). Addition of arundic acid (*P*<0.001) + valproic acid (0.5 mM, *P*<0.001) or valproic acid (1mM, *P*<0.001)) attenuated the intracellular S100B level compared to the Aβ-treated group. We summarized the results of intracellular S100B protein levels in Figure 2.

***Effects of different treatments on the extracellular S100B levels***

The present study showed that different treatments changed the extracellular S100B levels (F (7, 24) =1824.88 (7), *P*<0.001). The administration of Aβ elevated the extracellular S100B level in the astrocyte culture compared to the control group (*P*<0.001) (Figure 3). In contrast, arundic acid attenuated the extracellular S100B level compared to Aβ (*P*<0.001) and control (*P*<0.001) groups. Addition of arundic acid or valproic acid (0.5 and 1 mM) suppressed the extracellular S100B rising induced by Aβ (*P*<0.001). The astrocytes that received arundic acid + valproic acid + Aβ had a lower extracellular S100B level compared to Aβ and Aβ + arundic acid treated groups (*P*<0.001). We summarized the results of extracellular S100B protein levels in Figure 3. 

## Discussion

The present study showed that Aβ induced astrocytes apoptosis. In agreement, treatment of rat primary astrocyte cultures with Aβ decreased astrocyte viability (20). However, the concentrations of Saha and Biswas study (20) were much lower (4 µM) than the one used in our study mainly due to different cell types used. Other studies have reported the apoptotic action of Aβ on astrocytes (21-23). However, the mechanism of apoptotic action of Aβ on astrocytes needs to be elucidated. 

Astrocytes have critical functions in the CNS (24). Consequently, the impaired astrocytic functions may have possible roles in the pathophysiology of all neurological diseases (24). In this regard, damaged astrocytes have been demonstrated in major psychiatric disorders (25) and neurodegenerative disorders (26), particularly AD (3). Animal models have shown functional deficit of astrocytes and astrodegeneration in the brain regions implicated in the pathophysiology of AD (27-29). Reduced number or function of astrocytes may decrease metabolic support of neurons, impair neurotransmitter recycling, reduce Aβ clearance, and enhance Aβ-related neurodegeneration (15). Accordingly, glial dysfunction may be an initial manifestation of neurodegeneration (13, 26). Thus, astrodegeneration may be an important feature of AD and astroprotection may be a target to halt the progression of AD.

Our study showed that Aβ increased the apoptosis of astrocytes and raised the intracellular and extracellular S100B levels. S100B is mainly synthesized and released by astrocytes. Astrocytic activation induces S100B production and further enhances microglial activation and inflammatory responses (30). The severe inflammation may cause extensive injury in AD (31). Furthermore, overproduction of S100B contributed to the Aβ-induced neuritic changes in Down’s syndrome (32). Therefore, S100B may be associated with the pathogenesis of Aβ and progression of AD (33). Our results may imply that Aβ exposure increased the synthesis of S100B in the astrocytes. Moreover, apoptosis of astrocytes may increase the passive release of S100B to the extracellular space. Thus, the astrodegeneration induced by Aβ may be the cause of the elevated S100B level in the animal models and patients with AD. Moreover, protection of astrocytes against Aβ may attenuate the passive release of S100B into the extracellular space and diminish the inflammatory responses in AD. 

Recent evidence has shown the valproic acid ability to restore learning and memory deficit in the animal models of neurodegeneration (34, 35). Moreover, numerous *in vitro* and *in vivo* studies have shown neuro- and astroprotective properties of valproic acid against cytotoxic insults (36). Thus, valproic acid may be a potential drug for the treatment of AD (37). However, the effects of valproic acid and its mechanism of action in AD are not completely understood. Arundic acid is a new derivative of valproic acid that is under investigation for the treatment of neurodegenerative disorders (16). Arundic acid has decreased cerebral amyloidosis and glial activation in a transgenic model of AD (30). Our study showed that valproic and arundic acids protected astrocytes against Aβ toxicity. Arundic acid protected neurons against different stressors (18). However, there is limited evidence about the anti-apoptotic action of arundic acid on astrocytes. By considering homeostatic and neuroprotective functions of astrocytes, astroprotective effects of valproic and arundic acids may be partly responsible for the beneficial effects of these agents in AD.

Our study showed that Aβ increased S100B while arundic and valproic acids decreased the extracellular and intracellular S100B level. Our results may imply that the cytoprotective effects of valproic and arundic acids against Aβ may be related to the suppression of S100B production and secretion. According to the present study, Aβ increased both intracellular and extracellular S100B levels. In contrast, arundic and valproic acids decreased both intracellular and extracellular S100B levels. These results may imply that Aβ enhances synthesis and secretion of S100B while arundic and valproic acids reduce the synthesis and secretion of S100B from astrocytes. Previous reports have shown that arundic acid suppressed S100B synthesis in astrocytes (38). However, there are limited data about the effects of arundic acid on astrocyte protection. The high concentration of extracellular S100B produces cytotoxic effects (39) and enhances the neurotoxic effects of Aβ (40). Moreover, S100B level is up-regulated in AD (33) and suppression of S100B rising may affect the Aβ level and confer with AD-like pathology in the transgenic animals (30). Thus, drugs that modulate the S100B level may be potential candidates for the treatment of AD. 

Limitations

We evaluated the effects of arundic and valproic acids on astroprotection and S100B in an astrocytic cell line. For the extrapolation of these results, it seems necessary to confirm the results of this study in animal models. 

## Conclusion

Aβ induced astrocytic apoptosis and enhanced the S100B level. Arundic and valproic acids attenuated the S100B level and protected astrocytes against Aβ-induced toxicity. By considering homeostatic and neuroprotective functions of astrocytes, the astroprotective effects and the attenuation of S100B level may be responsible, at least in part, for the beneficial effects of these drugs in AD.
